# Endolysosome Iron Chelation Inhibits HIV-1 Protein-Induced Endolysosome De-Acidification-Induced Increases in Mitochondrial Fragmentation, Mitophagy, and Cell Death

**DOI:** 10.3390/cells11111811

**Published:** 2022-05-31

**Authors:** Peter W. Halcrow, Nirmal Kumar, Darius N. K. Quansah, Aparajita Baral, Braelyn Liang, Jonathan D. Geiger

**Affiliations:** Department of Biomedical Sciences, University of North Dakota School of Medicine and Health Sciences, 504 Hamline Street, Room 110, Grand Forks, ND 58203, USA; peter.halcrow@und.edu (P.W.H.); nirmal.kumar@und.edu (N.K.); darius.quansah@und.edu (D.N.K.Q.); aparajita.baral@und.edu (A.B.); braelyn.liang@und.edu (B.L.)

**Keywords:** endolysosomes, pH, mitochondria, autophagosomes, mitophagy, autophagy, deferoxamine

## Abstract

People with human immunodeficiency virus-1 (PLWH) experience high rates of HIV-1-associated neurocognitive disorders (HANDs); clinical symptoms range from being asymptomatic to experiencing HIV-associated dementia. Antiretroviral therapies have effectively prolonged the life expectancy related to PLWH; however, the prevalence of HANDs has increased. Implicated in the pathogenesis of HANDs are two HIV-1 proteins, transactivator of transcription (Tat) and gp120; both are neurotoxic and damage mitochondria. The thread-like morphological features of functional mitochondria become fragmented when levels of reactive oxygen species (ROS) increase, and ROS can be generated via Fenton-like chemistry in the presence of ferrous iron (Fe^2+^). Endolysosomes are central to iron trafficking in cells and contain readily releasable Fe^2+^ stores. However, it is unclear whether the endolysosome store is sufficient to account for insult-induced increases in levels of ROS, mitochondrial fragmentation, autophagy, and cell death. Using U87MG astrocytoma and SH-SY5Y neuroblastoma cells, we determined that chloroquine (CQ), Tat, and gp120 all (1) de-acidified endolysosomes, (2) decreased endolysosome numbers and increased endolysosome sizes, (3) increased mitochondrial numbers (fragmentation), (4) increased autophagosome numbers, (5) increased autolysosome numbers, (6) increased mitochondrial fragments within endolysosomes, and (7) increased cell death. These effects were all blocked by the endolysosome-specific iron chelator deferoxamine (DFO). Thus, the endolysosome de-acidification-induced release of endolysosome Fe^2+^ is sufficient to account for inter-organellar signaling events and cell biology consequences of HIV-1 proteins, including mitochondrial fragmentation, autophagy, and cell death.

## 1. Introduction

People living with human immunodeficiency virus-1 (PLWH) exhibit a high prevalence of HIV-1-associated neurocognitive disorders (HANDs); clinical symptomatology ranges from being “asymptomatic” to experiencing neurocognitive impairment and HIV-1-associated dementia [1]. Although antiretroviral therapies (ARTs) now effectively prolong life expectancy related to PLWH to near full lifespan, neurological complications persist [2]. Implicated in the pathogenesis of HANDs are HIV-1 virotoxic proteins including the transactivator of transcription (Tat) and gp120; they are neurotoxic, de-acidify endosomes and lysosomes (referred to as endolysosomes), and adversely affect mitochondria [3].

Both HIV-1 Tat and gp120 cause mitochondrial dysfunction and cell death [4,5,6,7]. Mitochondria are dynamic organelles that cycle between fission and fusion, processes that control their shape and numbers. Normal and functional mitochondria exhibit thread-like morphologies and insult-induced neurotoxicity results in mitochondrial fragmentation and impaired bioenergetics [8]. Increases in levels of reactive oxygen species (ROS) can cause mitochondrial fragmentation; HIV-1 Tat and gp120 increase ROS and mitochondrial fragmentation [3,9,10]. However, the upstream events and mechanisms by which HIV-1 Tat and gp120 induce these cytotoxic effects remain poorly understood.

ROS are generated through Fenton-like chemistry that requires ferrous iron (Fe^2+^). Endolysosomes are “master regulators of iron metabolism” [11,12,13] and contain readily releasable stores of Fe^2+^ sufficient to account for insult-induced releases of Fe^2+^ from endolysosomes and cytosolic and mitochondrial increases in ROS [14,15]. A distinguishing feature of endolysosomes is their acidic lumen and endolysosome de-acidification triggers iron dyshomeostasis [16]. Previously, we reported that HIV-1 Tat and gp120 de-acidify endolysosomes and that gp120-induced decreased levels of Fe^2+^ in endolysosomes result in increased levels of mitochondrial Fe^2+^ and ROS [15,17]. However, it remains unclear whether Tat- and gp120-induced increases in mitochondrial fragmentation are downstream of endolysosome stores of Fe^2+^.

Over the past decade, studies have increasingly noted the implications of autophagy, in particular the dysfunction of macroautophagy (hereafter referred to as autophagy), in the pathogenesis of various neurodegenerative conditions [18,19]. Autophagy is a highly dynamic and conserved pathway central to maintaining cellular homeostasis via the sequestration and delivery of damaged proteins and organelles into endolysosomes for degradation [20,21]. Endolysosomes are the degrading machine for autophagy, and endolysosome de-acidification agents such as chloroquine (CQ) inhibit the degradation capacity of endolysosomes [22]. Endolysosomes also affect autophagic activation, and CQ can activate autophagy, as seen by the formation of autophagosomes [22]. However, little is known about upstream events by which de-acidification agents induce autophagy and fragment mitochondria that become destined for endolysosome degradation (mitophagy).

Here, using U87MG astrocytoma cells as well as SH-SY5Y neuroblastoma cells, we showed that CQ, Tat, and gp120 (1) de-acidified endolysosomes, (2) decreased endolysosome numbers and increased endolysosome sizes, (3) increased the number of mitochondria, autophagosomes, fused autophagosomes–endolysosomes, and mitochondrial fragments within autophagosomes and endolysosomes, and (4) increased cell death, effects that were all blocked by the endocytosed iron chelator deferoxamine (DFO). In particular, DFO blocked CQ-, Tat-, and gp120-induced mitochondrial fragmentation and cell death. Thus, the CQ-, Tat-, and gp120-induced increases in mitochondrial fragmentation, mitophagic flux, and cell death appear to be downstream of endolysosome Fe^2+^ stores and the disruption of the endolysosome acidic lumen via de-acidification leading to iron dysregulation. Increased understanding of the role of endolysosome Fe^2+^ in HIV-1 protein-induced adverse effects may yield important new insights into the highly prevalent clinical condition known as HIV-1-associated neurocognitive disorder.

## 2. Materials and Methods

### 2.1. Cell Culture and Transfections

U87MG astrocytoma and SH-SY5Y neuroblastoma cells were cultured in Dulbecco’s Modified Eagle Medium (1× DMEM, Invitrogen, Waltham, MA, USA) containing 10% fetal bovine serum (FBS) and 1% penicillin/streptomycin solution (Invitrogen). They were grown in T75 (75 cm^2^ area) flasks and sub-cultured after reaching 80% to 90% confluence. Cells were passaged every 3 to 4 days using 0.025% trypsin (Invitrogen) and did not exceed 10 passages. Cells were maintained at 37 °C and 5% CO_2_. A cell line stably expressing LAMP1-RFP (Addgene, Watertown, MA, USA, #1817) was generated using U87MG cells and lipofectamine 2000 (Invitrogen, Waltham, MA, USA). Media was replaced 7 h after transfection, and incubation continued for 48 h. Expression levels were confirmed using confocal microscopy.

### 2.2. Reagents

HIV-1 recombinant Tat (#1002) and recombinant HIV-1 IIIB envelope glycoprotein gp120 (#1001) were purchased from ImmunoDX (Woburn, MA, USA). Chloroquine (cat no. C6628) and deferoxamine mesylate salt (cat no. D9533) were purchased from Sigma-Aldrich (St. Louis, MO, USA). Several aliquots were prepared and stored at −4 °C to prevent freeze–thaw problems. Mtphagy dye (cat no. MT02-10) was purchased from Dojindo Molecular Technologies, (Rockville, MD, USA).

### 2.3. Confocal Microscopy

Cells were seeded on 35 mm^2^ dishes at a density of 50,000 per 1 cm^2^ and allowed to attach overnight. After a total of 12 to 14 h, 2.5 μL/10,000 cells Premo-autophagy sensor LC3B-GFP (BacMam 2.0, cat no. P36235, Invitrogen, Waltham, MA, USA) was added, and cells were incubated for 18 h as per the company protocol. After 18 h, culture media was replaced with fresh media, and then, cells were treated with respective treatments for 24 h. After the completion of treatments, cells were gently washed twice with warm 1× PBS and re-suspended in 37 °C 1× PBS containing 50 nM of MitoTracker^TM^ Red FM (Thermo Fisher Scientific, Waltham, MA, USA, M22425) and 1 μg/mL Hoechst 33,342 (ThermoFisher) or 50 nM of LysoTracker^TM^ Far-Red DND-99 (Thermo Fisher Scientific, L7528) and 1 μg/mL Hoechst 33,342 (ThermoFisher) for 10 min in the dark at 37 °C and 5% CO_2_. Cells were washed three times with pre-warmed 1× PBS, and following the addition of fresh 1× PBS, images were taken with our spinning disk confocal microscope (Oxford Instruments, Concord, MA, USA). Images were analyzed using Imaris software (9.9.0 version). The numbers of endolysosomes (far-red puncta) and mitochondria (red puncta) were counted per cell. The formation and accumulation of autophagosomes (the number of colocalized with green and yellow fluorescence dots) were determined. All experiments were repeated at least three times.

### 2.4. Confocal Microscopy for Mitophagy Detection

Mitophagy was detected using a Mtphagy dye (mitophagy detection kit) as per the manufacturer’s protocol (Dojindo Molecular Technologies, Rockville, MD, USA). Mtphagy dye accumulates in intact mitochondria, and when mitophagy is induced, damaged mitochondria enter lysosomes and the Mtphagy dye emits fluorescence at an excitation of 530 nm and emission at 700 nm. U87MG cells (1 × 10^5^) were seeded on 35 mm^2^ dishes, and following incubation overnight at 37 °C and 5% CO_2_, cells were incubated at 37 °C and 5% CO_2_ with 0.1 nM of Mtphagy dye for 30 min. Cells were washed twice with warm 1× PBS, followed by the addition of fresh media. Cells were treated with respective treatments for 24 h. After 24 h, cells were washed gently with warm 1× PBS and re-suspended in 1× PBS containing 50 nM of LysoTracker^TM^ Green DND 26 (ThermoFisher Scientific, L7526) and 1 μg/mL Hoechst 33,342 (Invitrogen, c, H3570) for 10 min in the dark at 37 °C and 5% CO_2_. Next, cells were washed with 1× PBS three times, and mitophagy was observed with our spinning disk confocal microscope (Oxford Instruments, Concord, MA, USA) at an excitation of 530 nm and an emission of 700 nm. The acquired images were analyzed using Imaris software version 9.9.0. Mean fluorescence intensities (MFIs) and co-localization were quantified from 50 cells from each of three independent experiments.

### 2.5. Detection of Damaged and Intact Mitochondria

Mtphagy dye was used to detect damaged and intact mitochondria. U87MG cells were seeded in 24-well plates at a density of 1.0 × 10^5^ cells/well and were incubated overnight at 37 °C and 5% CO_2_. To detect damaged mitochondria, cells were stained with Mtphagy dye (0.1 nM) and incubated at 37 °C for 30 min. After 30 min, cells were washed twice with 1× PBS and supplemented with fresh media. Cells were treated for 24 h and washed twice with 1× PBS prior to being collected into separate Eppendorf tubes. Cells were analyzed with an Attune NxT flow cytometer (ThermoFisher, Waltham, MA, USA) using the FL2 channel. Flow cytometry data were collected from a minimum of 10,000 cells per condition. To detect intact mitochondria, cells were treated for 24 h and then incubated with Mtphagy dye (0.1 nM) for 30 min at 37 °C. Cells were washed, collected into separate Eppendorf tubes, and analyzed with an Attune NxT flow cytometer (ThermoFisher) using the FL2 channel. Flow cytometry data were collected from a minimum of 10,000 cells per condition.

### 2.6. Cell Death Assay

Cell viability was determined using a Propidium Iodide (PI) staining solution and flow cytometry according to the manufacturer’s protocol (MP Biomedicals, LLC, Irvine, CA, USA). U87MG cells were seeded at a density of 60,000 cells/well in 24-well plates, and cells were treated for 24 h. After 24 h, cells were collected into separate Eppendorf tubes and then centrifuged (5418 R, Eppendorf, Enfield, CT, USA) at 400× *g* for 10 min. Cell pellets were collected and resuspended in PI staining solution (3 µM of PI stain in PBS), mixed, and incubated at room temperature for 15 min in the dark. An Attune NxT Flow cytometer (ThermoFisher) with channel FL2 was used to detect cell death. Each sample was mixed properly before recording the response. Flow cytometry data were collected from a minimum of 20,000 cells per condition.

### 2.7. Statistics

All data were described as mean ± standard deviation (SD). Comparisons between treatment and control groups used either two-tailed Student’s *t*-tests or one-way ANOVA with appropriate post hoc tests. *p* values less than 0.05 were considered statistically significant. GraphPad Prism 9.3.1 software was used to perform all statistical analyses. No pre-determined exclusion criteria were performed. Normal distribution was assessed for statistical analysis by using the Shapiro–Wilk test (*p* < 0.05 was considered as not to conform to a normal distribution). The Kruskal–Wallis non-parametric test was used to compare data that had a skewed distribution. Results were based on experiments conducted at least three separate times, each time in triplicate.

## 3. Results

### 3.1. HIV-1 Tat- and gp120-Induced Endolysosome De-Acidification

**Figure 1:**
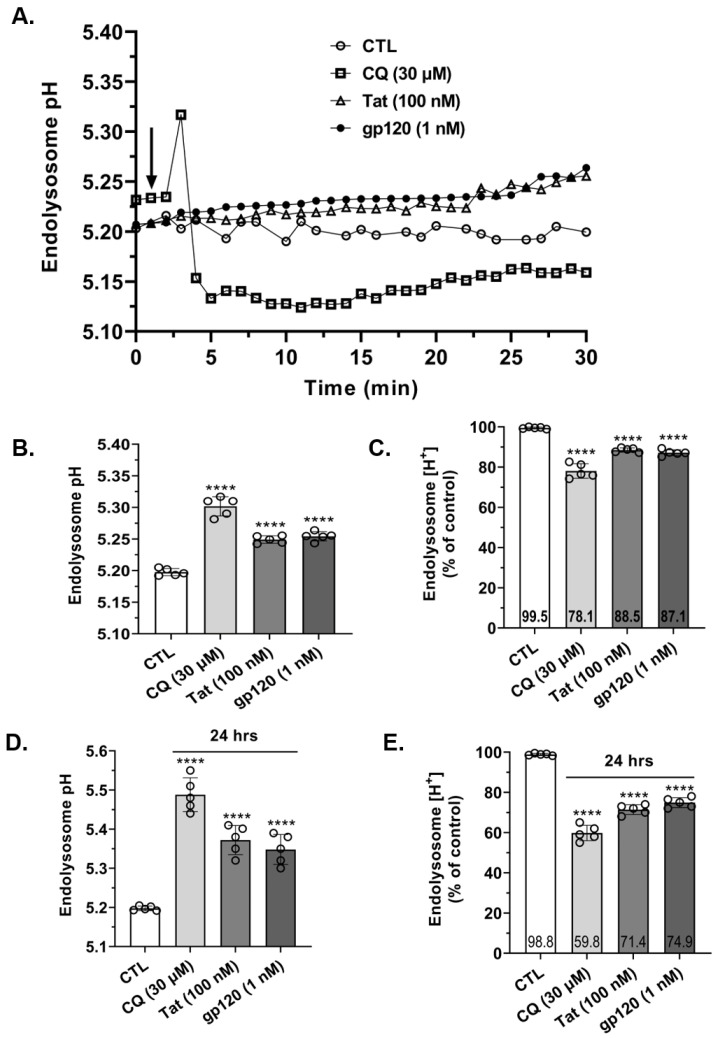
HIV-1 Tat, gp120, and CQ de-acidified endolysosomes. (**A**,**B**) Treatment of cells with CQ (30 µM), Tat (100 nM), and gp120 (1 nM) significantly (*p* < 0.0001) de-acidified endolysosome pH. Endolysosome de-acidification was shown in real time (arrowhead indicates the timepoint when each compound was added) (**A**) and as peak responses within 30 min (**B**). (**C**) Within 30 min, CQ (30 µM), Tat (100 nM), and gp120 (1 nM) significantly (*p* < 0.0001) decreased the H^+^ concentration of endolysosomes compared to controls. (**D**) At 24 h, CQ (30 µM), Tat (100 nM), and gp120 (1 nM) significantly (*p* < 0.0001) de-acidified endolysosome pH and (**E**) decreased [H^+^]. A one-way ANOVA with a Tukey’s post hoc multiple comparisons test was used for analysis. Each data point represents the mean pH measurement of 50 endolysosomes from five cells performed independently five times for each group (*n* = 250). Cells were chosen randomly in the microscope’s field of view with 25 cells used for experimentation per group; no cells were intentionally excluded. **** *p* < 0.0001.

Endolysosome de-acidification decreases concentrations of Fe^2+^ in endolysosomes ([Fe^2+^]_el_) [14,15]. Accordingly, we first determined the effects of HIV-1 Tat and gp120 and for comparison CQ on endolysosome pH. CQ (30 µM), Tat (100 nM), and gp120 (1 nM) significantly (*p* < 0.0001) de-acidified endolysosome pH; real-time (Figure 1A) and peak responses over a 30 min test period (Figure 1B) are illustrated. These changes in pH corresponded to decreases in endolysosome proton concentrations [H^+^] of 22% with CQ (30 µM), 12% with HIV-1 Tat (100 nM), and 13% with gp120 (1 nM) (Figure 1C). Because levels of HIV-1 are chronically elevated and CQ has an in vivo half-life of about 50 days [23], next, we determined their effects on endolysosome pH and [H^+^] at 24 h (Figure 1D,E). CQ (30 µM), Tat (100 nM), and gp120 (1 nM) significantly (*p* < 0.0001) de-acidified endolysosome pH at 24 h (Figure 1D), and these effects on pH corresponded to decreases in endolysosome [H^+^] of 40% for CQ (30 µM), 29% for Tat (100 nM), and 25% for gp120 (1 nM) (Figure 1E).

### 3.2. HIV-1 Tat- and gp120-Induced Increases in Mitochondrial Fragmentation Were Blocked by DFO

**Figure 2:**
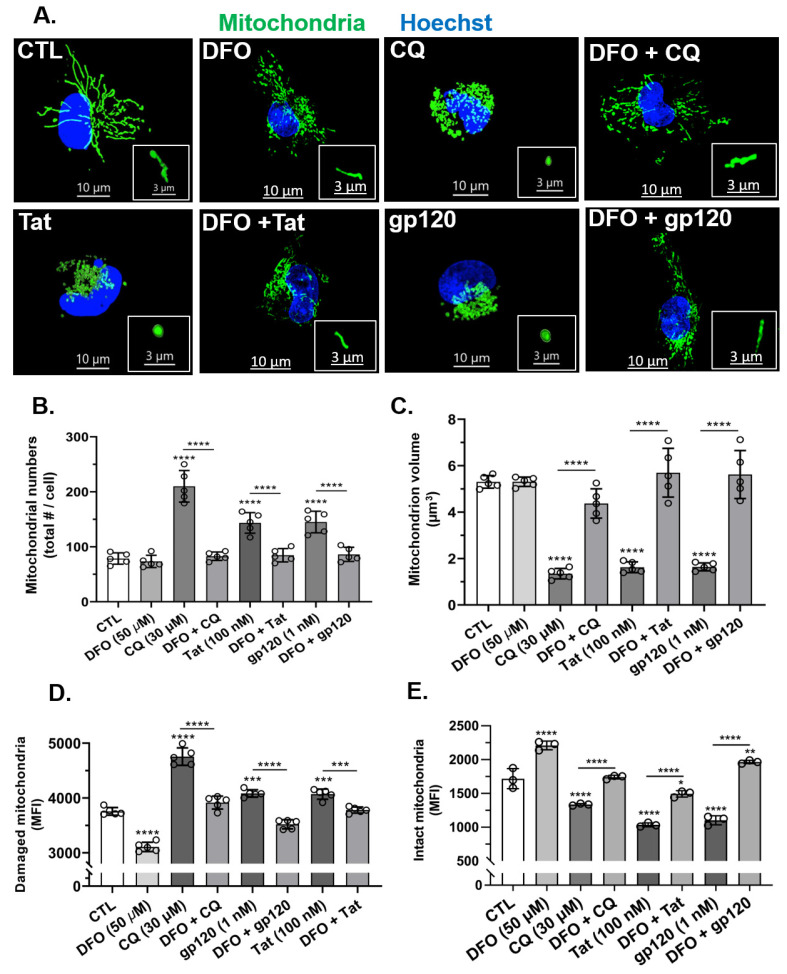
DFO blocked HIV-1 Tat-, gp120-, and CQ-induced increases in mitochondrial fragmentation and decreases in mitochondrial volume (size). (**A**) Representative fluorescence microscope images showing mitochondria (green) and nuclei (blue). DFO blocked CQ-, HIV-1 Tat-, and gp120-induced mitochondrial fragmentation (small and swelled structures) compared to control cells (elongated structures). (**B**,**C**) DFO blocked CQ-, HIV-1 Tat-, and gp120-induced mitochondrial fragmentation, increased mitochondrial numbers, and decreased mitochondrial volume. (**D**,**E**) DFO blocked CQ-, HIV-1 Tat-, and gp120-induced increases in damaged mitochondria and decreases in intact mitochondria. Image analysis and quantification was performed on five independent experiments with 30 cells quantified for each experimental condition (*n* = 150). Error bars represent standard deviation (SD) of five independent experiments. A one-way ANOVA multiple comparisons test was used to compare control group and treatment group. Cells were chosen randomly during image acquisition and analysis from the microscope field of view, and no cells were intentionally excluded. Scale bars are 10 µm for the image and 3 µm for the inset. * *p* < 0.05, ** *p* < 0.01, *** *p* < 0.001 and **** *p* < 0.0001.

Next, we determined the effects of HIV-1 Tat and gp120 on mitochondrial morphology, including numbers of mitochondria per cell, mitochondrion volume (size), mitochondrial damage, and mitochondrial intactness. HIV-1 Tat (100 nM), gp120 (1 nM), and CQ (30 µM) significantly (*p* < 0.0001) increased mitochondrial numbers and significantly (*p* < 0.0001) decreased mitochondrion volumes as compared to controls (Figure 2A–C). The pre-treatment of cells for 1 h with the endolysosome-specific iron chelator DFO (50 µM) significantly (*p* < 0.0001) blocked HIV-1 Tat-, gp120- and CQ-induced mitochondrial fragmentation (Figure 2A–C). To determine the extent to which HIV-1 Tat, gp120, and CQ damaged mitochondria, we used Mtphagy dye and initially showed that CQ increased the mean fluorescence intensity as damaged mitochondria entered acidic endolysosomes (Figure S1). Using this method, we were able to determine the levels of intact and damaged mitochondria. HIV-1 Tat (100 nM), gp120 (1 nM), and CQ (30 µM) significantly (*p* < 0.0001) increased MFI (mitochondrial damage) when the stain was added prior to treatment (Figure 2D) and significantly (*p* < 0.0001) decreased MFI (intact mitochondria) when the stain was added after the treatment (Figure 2E). The pre-treatment of cells for 1 h with DFO (50 µM) significantly (*p* < 0.0001) blocked HIV-1 Tat-, and gp120- and CQ-induced increases in damaged mitochondria (Figure 2D) and decreases in intact mitochondria (Figure 2E).

### 3.3. DFO Blocked HIV-1 Tat-, gp120- and CQ-Induced Increases in Autophagosome Numbers but Not Volumes

**Figure 3:**
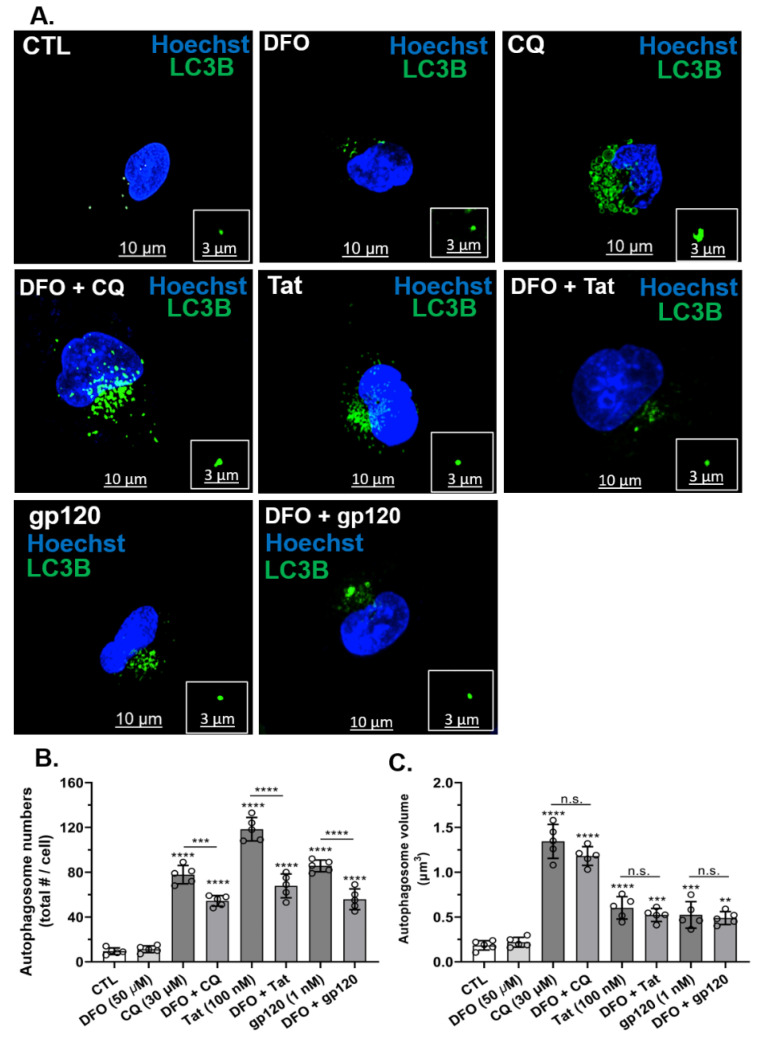
DFO blocked HIV-1 Tat-, gp120- and CQ-induced increases in autophagosome numbers but not volumes. (**A**) Representative fluorescence microcopy images of autophagosomes (LC3B, green) and nuclei (blue). Qualitatively, DFO blocked CQ-, HIV-1 Tat- and gp120-induced changes in autophagosome numbers and volume. (**B**,**C**) Quantitatively, CQ (30 µM), Tat (100 nM), and gp120 (1 nM) increased autophagosome numbers and volume, and DFO significantly (*p* < 0.0001) decreased autophagosome formation but did not reduce changes in autophagosome volumes. Image analysis and quantification were performed from five independent experiments with 30 cells quantified for each experimental condition (*n* = 150). Error bars represent standard deviation (SD) of five independent experiments. A one-way ANOVA multiple comparisons test was used to compare data from control and treatment groups. Cells were chosen randomly during image acquisition and analysis from the microscope field of view, and no cells were intentionally excluded. Scale bars are 10 µm for the image and 3 µm for the inset. ** *p* < 0.01, *** *p* < 0.001, and **** *p* < 0.0001; n.s.: non-significant.

Next, we determined the effects of HIV-1 Tat and gp120 as well as CQ on autophagosome numbers and volume. HIV-1 Tat (100 nM), gp120 (1 nM), and CQ (30 µM) significantly (*p* < 0.0001) increased the number of autophagosomes (Figure 3A,B) and autophagosome volume (Figure 3A,C) as compared to controls. The pre-treatment of cells for 1 h with DFO significantly (*p* < 0.0001) reduced HIV-1 Tat-, gp120-, and CQ-induced increases in the number of autophagosomes (Figure 3B). However, the pre-treatment of cells for 1 h with DFO did not significantly affect HIV-1 Tat-, gp120- and CQ-induced increases in autophagosome volume (Figure 3C).

### 3.4. DFO Blocked HIV-1 Tat-, gp120-, and CQ-Induced Increased Accumulation of Damaged Mitochondria in Autophagosomes

**Figure 4:**
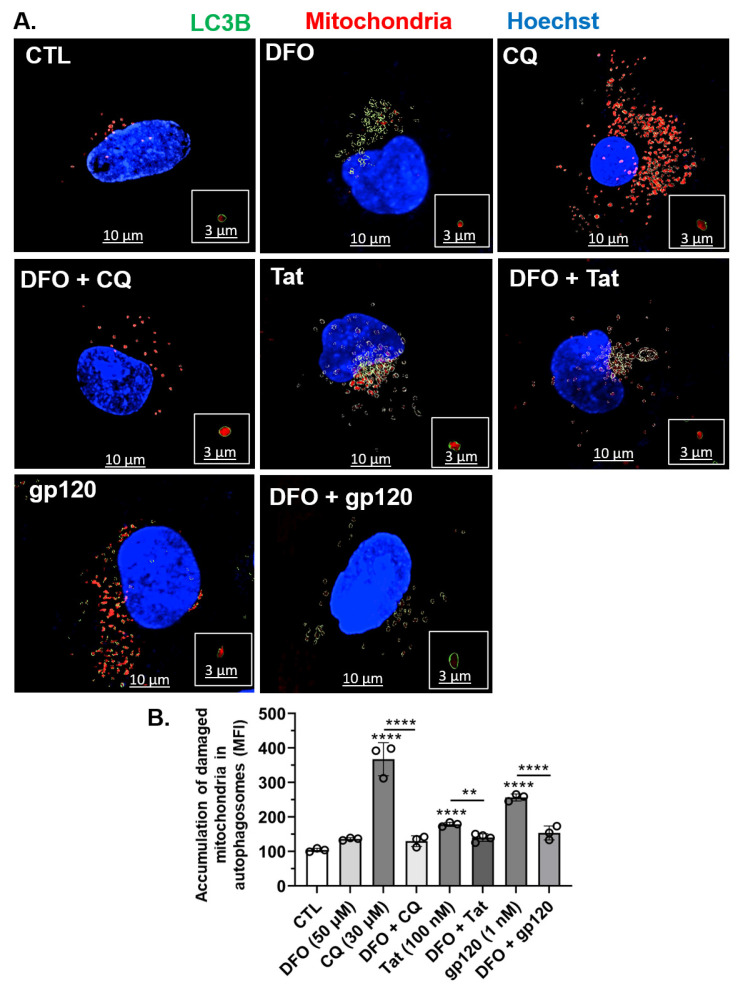
DFO blocked HIV-1 Tat-, gp120-, and CQ-induced accumulation of damaged mitochondria inside autophagosomes. (**A**) Imaris 3D reconstructed fluorescence microscopy images showing autophagosomes (green spherical structures), damaged mitochondria (red) inside autophagosomes (red inside green circle), and nuclei (blue). Qualitatively, DFO blocked CQ- (30 µM), Tat- (100 nM), and gp120- (1 nM) induced increases of mitochondria inside autophagosomes. (**B**) Quantitatively, DFO blocked CQ- (30 µM), Tat- (100 nM), and gp120- (1 nM) induced increases of mean fluorescence intensity (MFI) of mitochondria inside autophagosomes. Image analysis and quantification were performed from five independent experiments with 30 cells quantified for each experimental condition (*n* = 150). Error bars represent standard deviation (SD) of five independent experiments. A one-way ANOVA multiple comparisons test was used to compare control and treatment groups. Cells were chosen randomly during image acquisition and analysis from the microscope field of view, and no cells were intentionally excluded. Scale bars are 10 µm for the image and 3 µm for the inset. ** *p* < 0.01 and **** *p* < 0.0001.

Next, using U87MG cells, we determined whether Tat-, gp120- and CQ-induced increases in mitochondrial damage led to the accumulation of damaged mitochondrial in autophagosomes. CQ (30 µM) and to a lesser extent HIV-1 Tat (100 nM) and gp120 (1 nM) significantly (*p* < 0.0001) increased the accumulation of mitochondrial fragments within autophagosomes as compared to controls (Figure 4A,B). The pre-treatment of cells for 1 h with DFO (50 µM) resulted in the statistically significant (*p* < 0.0001) reversal of the increased accumulation of damaged mitochondria in autophagosomes induced by HIV-1 Tat, gp120, and CQ (Figure 4A,B).

### 3.5. HIV-1 Tat, gp120, and CQ Decreased Endolysosome Numbers and Increased Endolysosome Volumes

**Figure 5:**
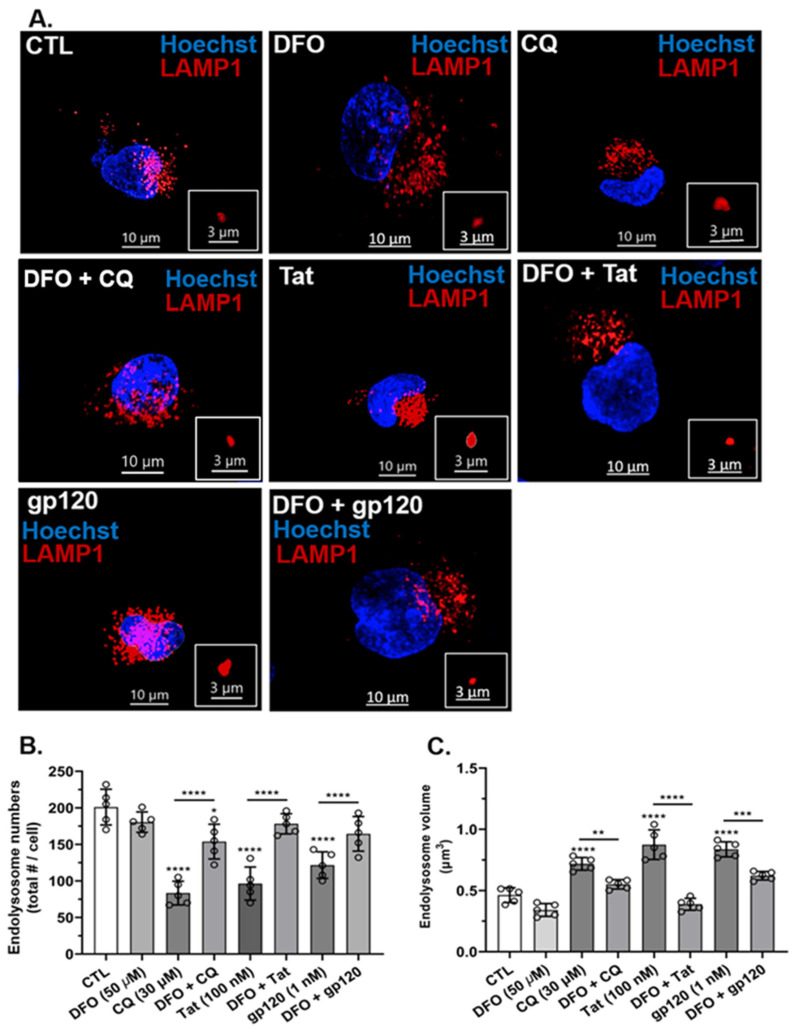
DFO blocked HIV-1 Tat-, gp120-, and CQ-induced decreases in endolysosome numbers and increases in endolysosome volume. (**A**) Representative fluorescence microscopy images showing endolysosomes (red puncta) and nuclei (blue) and that DFO blocked CQ- (30 µM), Tat- (100 nM), and gp120- (1 nM) induced decreases in endolysosome numbers and increases in endolysosome volume. (**B**,**C**) Quantitatively, DFO blocked CQ-, Tat-, and gp120-induced decreases in endolysosome numbers and increases in endolysosome volume. Image analysis and quantification were performed from five independent experiments with 30 cells quantified for each experimental condition (*n* = 150). Error bars represent standard deviation (SD) of five independent experiments. A one-way ANOVA multiple comparisons test was used to compare control group and treatment group. Cells were chosen randomly during image acquisition and analysis from the microscope field of view, and no cells were intentionally excluded. Scale bars are 10 µm for the image and 3 µm for the inset. * *p* < 0.05, ** *p* < 0.01, *** *p* < 0.001 and **** *p* < 0.0001.

Endolysosome activity is strongly dependent on their numbers and volumes [24]. Using U87MG cells, next, we determined effects of HIV-1 Tat, gp120, and CQ on endolysosome numbers per cell and endolysosome volumes. The treatment of cells for 24 h with HIV-1 Tat (100 nM), gp120 (1 nM), and CQ (30 µM) significantly (*p* < 0.0001) decreased the numbers of endolysosomes per cell as compared to controls (Figure 5A,B) and significantly (*p* < 0.0001) increased endolysosome volumes (size) as compared to controls (Figure 5A,C). Pre-treatment for 1 h with DFO significantly (*p* < 0.0001) blocked CQ-, Tat-, and gp120-induced decreases in the number of endolysosomes per cell and blocked the increase in endolysosome volume when comparing the control and treated groups (Figure 5B,C).

### 3.6. DFO Increased HIV-1 Tat-, gp120-, and CQ-Induced Increased Fusion between Endolysosomes and Autophagosomes

**Figure 6:**
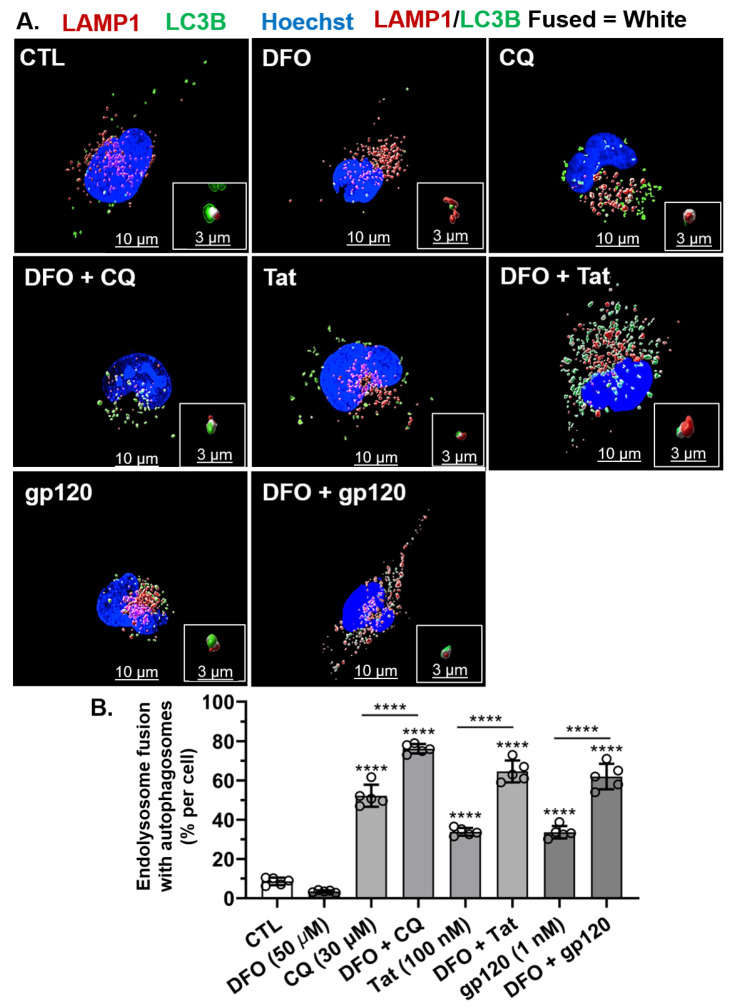
DFO increased HIV-1 Tat-, gp120-, and CQ-induced increases in endolysosome fusion with autolysosomes. (**A**) Representative fluorescence microscopy reconstructed (using Imaris 3D software) images showing autophagosomes (green puncta), endolysosomes (red puncta), endolysosomes fused with autolysosomes (white puncta), and nuclei (blue). Qualitatively, DFO increased autophagosome fusion with endolysosomes induced by CQ (30 µM), Tat (100 nM), and gp120 (1 nM). (**B**) Quantitatively, DFO increased the percentage of endolysosomes fusing with autophagosomes per cell induced significantly (*p* < 0.0001) by CQ (30 µM), Tat (100 nM), and gp120 (1 nM). Image analysis and quantification was performed from five independent experiments with 30 cells quantified for each experimental condition (*n* = 150). Error bars represent standard deviation (SD) of five independent experiments. A one-way ANOVA multiple comparisons test was used to compare the control group and treatment groups. Cells were chosen randomly during image acquisition and analysis from the microscope field of view, and no cells were intentionally excluded. Scale bars are 10 µm for the image and 3 µm for the inset. **** *p* < 0.0001.

Next, we determined the effects of HIV-1 Tat, gp120, and CQ on fusion between autophagosomes and endolysosomes to form autolysosomes and the ability of DFO to affect those changes. HIV-1 Tat (100 nM), gp120 (1 nM), and CQ (30 µM) significantly (*p* < 0.0001) increased the fusion between endolysosomes and autophagosomes; these treatments increased the number of autolysosomes per cell as compared to controls (Figure 6A,B). Pre-treatment for 1 h with DFO significantly (*p* < 0.0001) increased the percentage of endolysosomes fused with autophagosomes induced by CQ, Tat, and gp120 treatments. This potentiation of the effects of HIV-1 Tat, gp120 and CQ may have resulted from DFO-induced increases in endolysosome numbers, as illustrated in Figure 5B, and/or DFO-induced decreases in autophagosome numbers, as illustrated in Figure 3B.

### 3.7. DFO Blocked HIV-1 Tat-, gp120-, and CQ-Induced Increases in the Accumulation of Damaged Mitochondria in Endolysosomes

**Figure 7:**
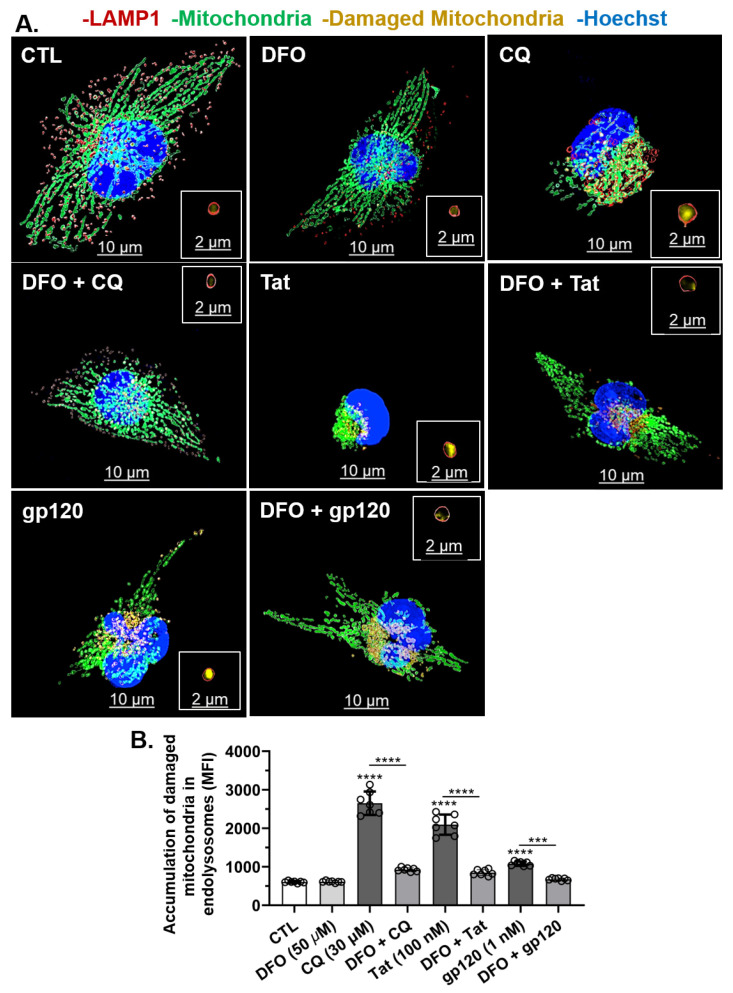
DFO blocked HIV-1 Tat-, gp120-, and CQ-induced increases in the accumulation of damaged mitochondrial fragments inside endolysosomes. (**A**) Representative fluorescence microscopy reconstructed (using Imaris 3D software) images showed late endosome–early lysosome (endolysosome) LAMP-1 RFP (red puncta), mitochondria (CellLight transfection, green), damaged mitochondrial fragments inside endolysosome (yellow puncta) and nuclei (blue). Qualitatively, HIV-1 Tat, gp120, and CQ increased the accumulation of damaged mitochondria fragments inside endolysosomes, and DFO blocked these increases. (**B**) Quantitatively, DFO blocked the accumulation of damaged mitochondrial fragments inside endolysosomes increased by CQ (30 µM), Tat (100 nM), and gp120 (1 nM). Image analysis and quantification was performed from five independent experiments with 30 cells quantified for each experimental condition (*n* = 150). Error bars represent standard deviation (SD) of five independent experiments. A one-way ANOVA multiple comparisons test was used to compare control and treatment groups. Cells were chosen randomly during image acquisition and analysis from the microscope field of view, and no cells were intentionally excluded. Scale bars are 10 µm for the image and 2 µm for the inset. *** *p* < 0.001 and **** *p* < 0.0001.

**Figure 8:**
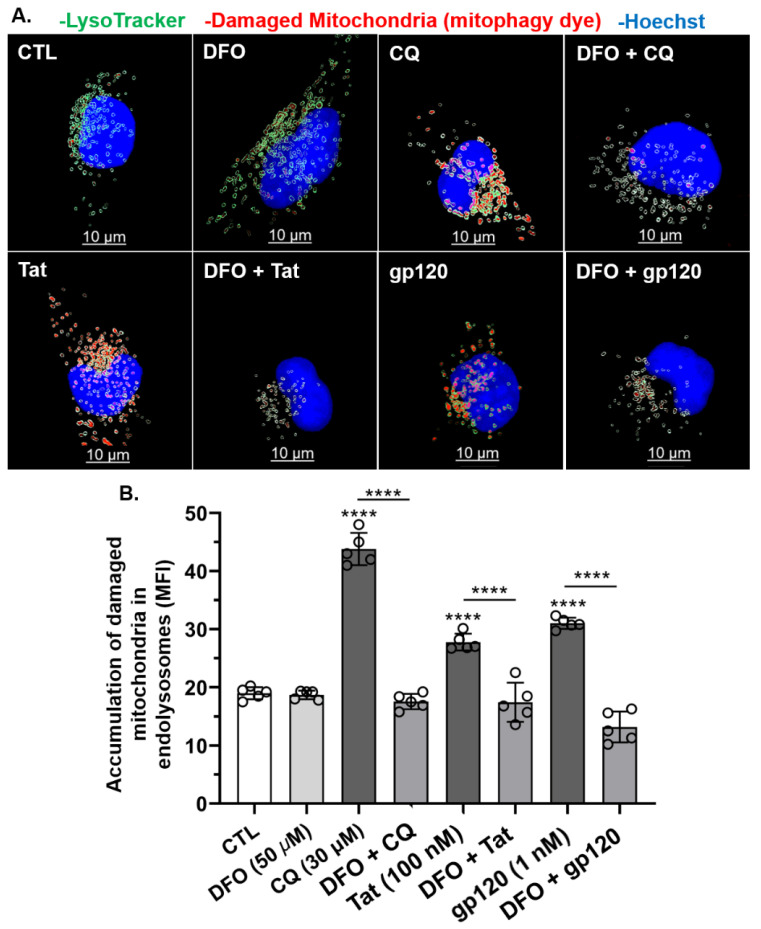
DFO blocked HIV-1 Tat-, gp120-, and CQ-induced increases in the accumulation of damaged mitochondria inside endolysosomes. (**A**) Representative fluorescence microscopy reconstructed (using Imaris 3D software) images showing endolysosomes (green), damaged mitochondria (red) inside endolysosomes (red inside green puncta), and nuclei (blue). Qualitatively, DFO blocked the accumulation of damaged mitochondria in endolysosomes induced by CQ (30 µM), Tat (100 nM), and gp120 (1 nM). (**B**) Quantitatively, DFO significantly blocked the accumulation of damaged mitochondria inside endolysosomes increased by CQ (30 µM), Tat (100 nM), and gp120 (1 nM) (**** *p* < 0.00001). Image analysis and quantification were performed from five independent experiments with 30 cells quantified for each experimental condition (*n* = 150). Error bars represent standard deviation (SD) of five independent experiments. A one-way ANOVA multiple comparisons test was used to compare control and treatment groups. Cells were chosen randomly during image acquisition and analysis from the microscope field of view, and no cells were intentionally excluded. Scale bar is 10 µm. **** *p* < 0.00001.

Next, we investigated whether HIV-1 Tat-, gp120-, and CQ-induced mitochondrial damage led to the accumulation of damaged mitochondria in endolysosomes of U87MG cells. HIV-1 Tat (100 nM), gp120 (1 nM), and CQ (30 µM) significantly (*p* < 0.0001) increased the accumulation of damaged mitochondrial fragments within endolysosomes as compared to controls (Figure 7A,B). Pre-treatment for 1 h with DFO significantly (*p* < 0.0001) blocked the accumulation of damaged mitochondria in endolysosomes induced by HIV-1 Tat, gp120, and CQ (Figure 7A,B). These findings were further validated using the mitophagy-specific fluorescence-based dye (Mtphagy). In U87MG cells stained with Mtphagy dye for 30 min, HIV-1 Tat (100 nM), gp120 (1 nM), and CQ (100 nM) significantly (*p* < 0.0001) increased the accumulation of damaged mitochondria in endolysosomes as compared to controls at 24 h (Figure 8A,B). The pre-treatment of cells for 1 h with DFO (50 µM) significantly (*p* < 0.0001) blocked the HIV-1 Tat-, gp120-, and CQ-induced increases in damaged mitochondria in endolysosomes (Figure 8B).

### 3.8. DFO Blocked HIV-1 Tat-, gp120-, and CQ-Induced Cell Death

**Figure 9:**
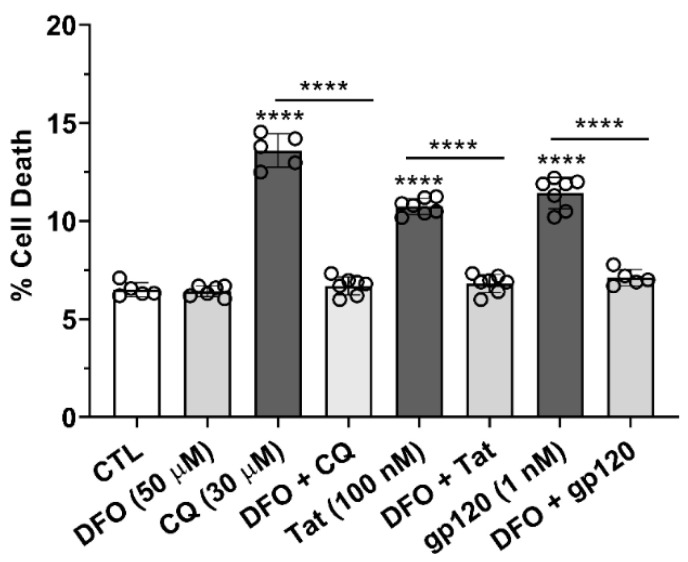
DFO blocked HIV-1 Tat- and gp120-induced cell death. Propidium iodide was used to measure cell death on U87MG cells after 24 h of treatment with CQ (30 µM), Tat (100 nM), and gp120 (1 nM). Cell death was significantly (*p* < 0.0001) increased by Tat (100 nM), gp120 (1 nM), and CQ (30 µM); 1 h pre-treatment with DFO (50 µM) blocked CQ-, Tat-, and gp120-induced cell death. A one-way ANOVA with a Tukey’s post hoc multiple comparisons test was used for analysis. Each data point represents the mean fluorescence from 20,000 cells performed independently at least five times for each group (*n* = 100,000). *n* = total number of cells for each group plotted. **** *p* < 0.0001.

Cell viability was determined using propidium iodide 24 h after the addition of HIV-1 Tat (100 nM), gp120 (1 nM), and CQ (30 µM). HIV-1 Tat, gp120, and CQ significantly (*p* < 0.0001) increased cell death as compared to controls (Figure 9). Pre-treatment for 1 h with DFO significantly (*p* < 0.0001) blocked HIV-1 Tat-, gp120-, and CQ-induced cell death (Figure 9).

## 4. Discussion

People living with HIV-1 who take effective antiretroviral therapeutics are now living almost full life spans but experience a high prevalence of HANDs, which deteriorate their quality of life [1]. Implicated in the pathogenesis of HANDs are the HIV-1 viral proteins Tat and gp120; both are neurotoxic, increase levels of ROS, induce mitochondria dysfunction and mitophagy, and cause neural cell death [5,25].

Endolysosomes are acidic organelles known for their physiological importance and pathological relevance [26,27,28,29]. Endolysosomes contain high levels of divalent cations including Fe^2+^, and these endolysosome iron stores are central to iron trafficking and redox signaling [13,14,30]. Iron homeostasis is linked to endolysosome acidity, whereupon endolysosome de-acidification triggers iron dysregulation. Fe^2+^ levels in endolysosomes are sufficient to affect Fe^2+^ levels in cytosol and in mitochondria. Indeed, the gp120-induced de-acidification of endolysosomes decreased Fe^2+^ levels in endolysosomes, increased Fe^2+^ levels in cytosol and mitochondria, increased levels of ROS in cytosol and mitochondria, and increased cell death [16,31]. Further, those neurotoxic effects were all blocked by the addition of the endolysosome-specific iron chelator deferoxamine; these are findings that indicate that the endolysosome pool of Fe^2+^ is sufficient to account for those downstream events. Because Fe^2+^ generates ROS via Fenton-like chemistry and increased levels of ROS can damage mitochondria and induce cell death [8,32,33], it was important to determine the extent to which the endolysosome pool of Fe^2+^ was sufficient to account for HIV-1 Tat-, gp120-, and for comparison, CQ-induced effects on downstream events including mitochondrial fragmentation, mitophagy, and cell death.

Principally, we found that HIV-1 Tat, gp120, and CQ de-acidified endolysosomes, decreased numbers of endolysosomes yet increased endolysosome size, increased the number and size of autophagosomes, increased the number of mitochondria via mitochondrial fragmentation, increased the number of autolysosomes, increased damaged mitochondrial fragments within endolysosomes, and induced cell death, effects that were all blocked by the endolysosome-specific iron chelator DFO. Thus, those effects appear to be downstream of endolysosome de-acidification and an efflux of the readily releasable store of endolysosome Fe^2+^.

Autophagy is a highly dynamic and conserved pathway that is central to maintaining cellular homeostasis via the sequestration and delivery of damaged proteins and organelles to endolysosomes where they are degraded; increasingly, autophagy dysfunction has been linked to the pathogenesis of various neurodegenerative diseases [18,19]. Endolysosome de-acidification via agents such as CQ affects the degradative and autophagic abilities of endolysosomes [22]; CQ inhibited autophagic degradation, increased the formation of autophagosomes, and activated autophagy [22]. However, relatively little is known about how endolysosome de-acidification agents induce autophagy and disrupt thread-like functional mitochondria via fragmentation that undergo mitophagy and degradation inside endolysosomes. Because of the close connection between endolysosome dysfunction and neurodegeneration [34,35,36], our findings that the endolysosome de-acidification-induced release of endolysosome iron caused increases in mitochondria ROS and fragmentation as well as autophagy (mitophagy) suggest that endolysosomes function to maintain cellular homeostasis through the enhanced degradation of damaged mitochondria.

We and others have shown that CQ, HIV-1 Tat, and gp120 de-acidify endolysosomes and cause an efflux of cations from endolysosomes sufficient to induce cytotoxicity [15,37,38,39]. Specifically, the endolysosome de-acidification-induced release of Fe^2+^ from endolysosomes was sufficient to increase Fe^2+^ and ROS levels in cytosol and mitochondria and cause mitochondrial damage. Here, we examined the effects of CQ, HIV-1 Tat, and gp120 on the number and sizes of endolysosomes, autophagosomes, and mitochondria at treatment intervals of 24 h because CQ has a very long half-life in humans [23] and HIV-1 proteins can remain elevated for extended periods of time in PLWH [40,41,42]. At 24 h, endolysosome enzyme activity was at its lowest level [22] and CQ, HIV-1 Tat, and gp120 decreased the numbers of endolysosomes yet increased their size, increased the numbers and sizes of autophagosomes, and increased the number of mitochondria due to fragmentation. Increased numbers of autophagosomes are indicative of cytotoxicity [43,44,45] and may result from insult-induced decreases in the number of endolysosomes [44]. Additionally, in agreement with previous studies [3,25], here, we observed that HIV-1 Tat and gp120 increased mitochondrial fragmentation and increased the number of separate and independent mitochondria. One of the known initiating factors that induces mitochondria fragmentation is ROS [8,32]. Consistent with findings from previous studies of others and ourselves, Tat and gp120 increase levels of mitochondrial ROS. Here, we report that HIV-1 proteins induce the fragmentation of thread-like mitochondria into many smaller separate mitochondria [3,15,37].

We also found that CQ, HIV-1 Tat, and gp120 damaged mitochondria, decreased numbers of intact mitochondria, increased mitochondria within endolysosomes and autophagosomes, and increased endolysosome–autophagosome fusion (autolysosome). Endolysosome degradation is dependent on an acidic lumen [46]; CQ, Tat, and gp120 significantly increase endolysosome pH, and the de-acidification of endolysosomes disrupts autophagic flux [22,37,47,48]. Thus, the insult-induced disruption in autophagic flux appears to be more related to the de-acidification-induced inhibition of endolysosome degradation than the inhibition of fusion between autophagosomes and endolysosomes.

**Figure 10:**
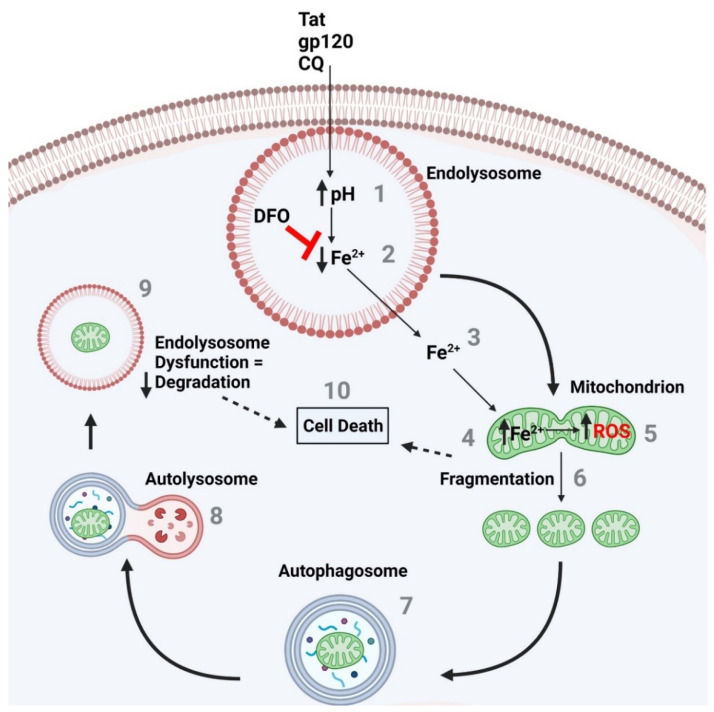
The endolysosome ferrous iron efflux induced by CQ-, Tat-, and gp120-induced de-acidification of endolysosomes increases mitochondrial ROS and fragmentation, autophagy activation, and increased cell death. (**1**) CQ and the HIV-1 proteins Tat and gp120 increased endolysosome pH, which is known to (**2**) induce a decrease in endolysosome Fe^2+^ levels and (**3**) an efflux in Fe^2+^ from endolysosomes into the cytosol and (**4**) an increase in mitochondrial Fe^2+^ and (**5**) ROS levels. The effects on mitochondrial ROS levels result in (**6**) the fragmentation of mitochondria and the (**7**) activation of autophagy, as seen by the formation of autophagosomes and (**8**) the fusion of autophagosomes with endolysosomes (autolysosomes). (**9**) Damaged mitochondria then appear in endolysosomes for degradation; however, the endolysosome pH is elevated, and as demonstrated previously, inactive degradation enzymes due to an elevated pH means the endolysosome enzymes are not degrading the damaged mitochondria as needed. (**10**) This may lead to cell death. In addition, the fragmented mitochondria result in a decrease in bioenergetics and may result in cell death as well. The endolysosome-specific iron chelator deferoxamine DFO blocks the CQ-, Tat-, and gp120-induced endolysosome iron efflux, the increase in mitochondrial iron and ROS levels, the increase in fragmentation, as well as the increase in cell death.

The formation of ROS is iron-based, and cell death can be caused by increases in oxidative damage and mitochondrial dysfunction [49]. Others and ourselves have shown that the chelation of endolysosome stores of iron with DFO can block CQ-, HIV-1 Tat-, and gp120-induced mitochondrial fragmentation and cell death. The rescue of ROS-induced cell death by ROS scavengers has been previously demonstrated [50]. DFO is known to acidify endolysosomes, chelate iron, and decrease levels of ROS [51]. A combination of these effects likely explains the protective actions of DFO against HIV-1 protein-induced cell death. Thus, the stores of endolysosome Fe^2+^ releasable by endolysosome de-acidification appear to be sufficient to account for insult-induced mitochondrial dysfunction and cell death. Endolysosome dysfunction appears to be an early event, with the release of its Fe^2+^ stores causing mitochondrial damage and initiating autophagy, yet the result is damaged mitochondria being delivered to endolysosomes for degradation, where there is a decrease in the enzyme activity of these degradation machines (Figure 10). Thus, endolysosome the de-acidification-induced inter-organellar signaling of endolysosome Fe^2+^ may be key to understanding CQ-, Tat-, and gp120-induced adverse effects, including damaged mitochondria, cell death, and cytotoxicity.

A few limitations to this study are worthy of comment. First, only DFO was used to chelate iron and reduce ROS levels. Second, we used only SH-SY5Y cells and U87MG astrocytoma cells; thus, our findings would benefit from replication in primary cultured neural cells. Third, cell death was only assessed using PI staining, and it would be interesting to further investigate the mechanisms by which cell death occurred. Finally, it would be interesting to determine the extent to which various ROS scavengers could block HIV-1 protein-induced cell death. These limitations can be addressed in future studies.

## Data Availability

Not applicable.

## Supplementary Materials

The following supporting information can be downloaded at: https://www.mdpi.com/article/10.3390/cells11111811/s1, Figure S1: Mtphagy dye binds intact mitochondria and fluorescence intensity increases when damaged mitochondria enter acidic endolysosomes.
