# Supplementary material for: Endolysosome Iron Chelation Inhibits HIV-1 Protein-Induced Endolysosome De-Acidification-Induced Increases in Mitochondrial Fragmentation, Mitophagy, and Cell Death
Source: Cells. 2022 May 31;11(11):1811. doi: 10.3390/cells11111811 (PMC9180803; doi:10.3390/cells11111811)
Supplement: Supplementary file 1 [file cells-11-01811-s001.zip › cells-1722345-supplementary.pdf]

cells

MDPI

Article

Endolysosome Iron Chelation Inhibits HIV-1 Protein-Induced Endolysosome De-Acidification-Induced Increases in Mito-chondrial Fragmentation, Mitophagy, and Cell Death

Supplementary Materials

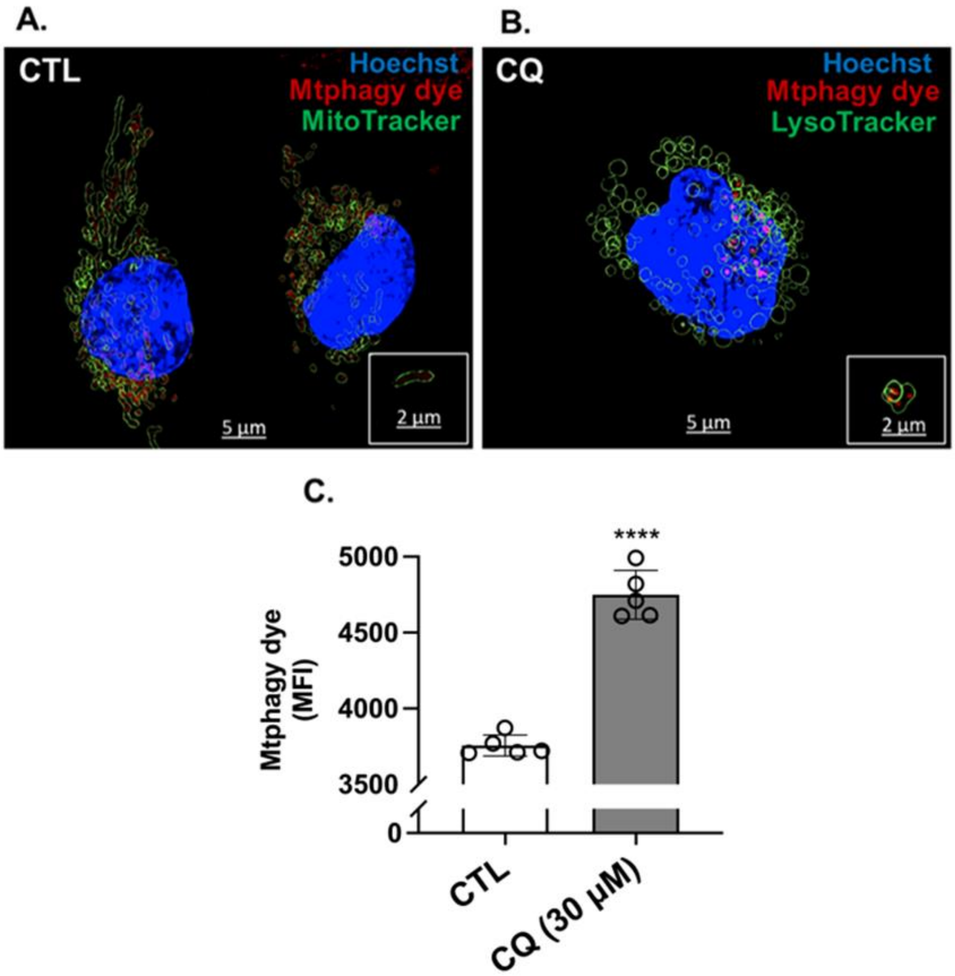

Figure S1. Mtphagy dye binds intact mitochondria and fluorescence intensity increases when dam-aged mitochondria enter acidic endolysosomes. (**A**) Representative fluorescence microscopy images were reconstructed using Imaris 3D software and in control cells illustrate mitochondria (green out-lines), intact mitochondrial Mtphagy staining (dim red fluorescence inside green outlined orga-nelles), and the nucleus (blue). (**B**) Treatment of cells with CQ (30 µM) for 24 h significantly ($p < 0.0001$) increased the Mtphagy dye fluorescence in endolysosomes. (**C**) Quantification of intact and damaged mitochondrial staining demonstrated that CQ (30 µM) significantly ($p < 0.0001$) increased mean fluorescence intensity (MFI) compared to controls. Error bars represent standard deviation (SD) of five independent experiments ($n = 150$). A Student's t-test was used for analysis to compare treatment with controls. Cells were chosen randomly during image acquisition and no cells were intentionally excluded. Scale bars are 5 µm for the images and 2 µm for the insets.
